# Crown Work

**Published:** 1885-08

**Authors:** M. W. Williams

**Affiliations:** Hopkinsville, Ky.


### Crown Work.

BY M. W. WILLIAMS, HOPKINSVILLE, KY.

Profitable and popular crown work must be done in short time, with ease, both to operator and patient, must be durable and artistic. This ideal has as yet not been attained by Howe, Bonwell, Richmond, Logan, or Sheffield, as many will attest. But that I have conquered part of the field, you are only to see to be convinced, by part of the field, I mean to say for incisors and canines can cry "*Eureka*"--I have complied with every condition mentioned above. I will give a short pen sketch, omitting details.

The crown is similar to old-fashion wooden pivot crown, except the pivot-hole is lined with platina tube. A similar tube ¼-inch long, with bottom and depending shank of platina securely soldered to it designed to be secured in root canal. The lower portion of this tube is slightly larger than orifice. There is solid platina connecting post, in size easily to pass in tubes, in length so that mouths of tubes will meet when in position. The lower ¼-inch of post is slitted for reception of key wedge which is a small tapering steel wedge ¼-inch long and thickness of No. 1 file at its thickest end.

To adjust it, grind off root on line with gum, enlarge canal, so that tube and its depending shank will drop in loosely beyond level with end of root. Grind base of crown to approximate joint with end of root. Solder unslitted end of post in tube of crown, slip tube of root attachment over slitted end; mix agate cement thin, fill canal one-half full, force all in position, have the patient close the mouth, hold steady in articulating position until cement hardens; withdraw crown and post, leaving root attachment in exact position; trim down the cement, fill out with amalgam, allowing it to spread thinly, all over the end of root; insert small key wedge; place firm piece of wood on cutting edge of crown, with mallet gently drive it home; the end of the key striking the bottom of the tube will force itself in expanding sides of post to fill the dovetailed space. Thus you can readily see it is impossible for it ever to become loosened or detached. Advantages. Its simplicity, strength and durability; strongest because of the size and shortness of the post, and having more procelain, strengthen by tube, in crown. The crown being out of the way. The ease of perfectly forcing amalgam round root attachment in canal, and efficiency in which the end of root can be protected from further decay. *It is self-articulating.* No exposure of cement, and but a line of amalgam at the gum margin. It is artistic in that it conceals art; can be permanently secured at one short sitting of thirty minutes without causing your patient any pain, and with the greatest comfort to yourself, knowing it will remain as long as the root stays in the mouth. One other advantage in case of accident to the porcelain, is the great ease with which another crown can be attached.

